# A first generation BAC-based physical map of the half-smooth tongue sole (*Cynoglossus semilaevis*) genome

**DOI:** 10.1186/1471-2164-15-215

**Published:** 2014-03-20

**Authors:** Junjie Zhang, Changwei Shao, Liyan Zhang, Kun Liu, Fengtao Gao, Zhongdian Dong, Peng Xu, Songlin Chen

**Affiliations:** 1 Yellow Sea Fisheries Research Institute, Chinese Academy of Fishery Sciences, Qingdao 266071, China; 2 College of Fisheries and Life Science, Shanghai Ocean University, Shanghai 201306, China; 3 College of Animal Science, Xinjiang Agricultural University, Urumqi 830052, China; 4 The Centre for Applied Aquatic Genomics, Chinese Academy of Fishery Sciences, Beijing 100141, China

**Keywords:** Half-smooth tongue sole, *Cynoglossus semilaevis*, BAC library, Physical map, Genome

## Abstract

**Background:**

Half-smooth tongue sole (*Cynoglossus semilaevis* Günther) has been exploited as a commercially important cultured marine flatfish, and female grows 2–3 times faster than male. Genetic studies, especially on the chromosomal sex-determining system of this species, have been carried out in the last decade. Although the genome of half-smooth tongue sole was relatively small (626.9 Mb), there are still some difficulties in the high-quality assembly of the next generation genome sequencing reads without the assistance of a physical map, especially for the W chromosome of this fish due to abundance of repetitive sequences. The objective of this study is to construct a bacterial artificial chromosome (BAC)-based physical map for half-smooth tongue sole with the method of high information content fingerprinting (HICF).

**Results:**

A physical map of half-smooth tongue sole was constructed with 30, 294 valid fingerprints (7.5 × genome coverage) with a tolerance of 4 and an initial cutoff of 1e-60. A total of 29,709 clones were assembled into 1,485 contigs with an average length of 539 kb and a N50 length of 664 kb. There were 394 contigs longer than the N50 length, and these contigs will be a useful resource for future integration with linkage map and whole genome sequence assembly. The estimated physical length of the assembled contigs was 797 Mb, representing approximately 1.27 coverage of the half-smooth tongue sole genome. The largest contig contained 410 BAC clones with a physical length of 3.48 Mb. Almost all of the 676 BAC clones (99.9%) in the 21 randomly selected contigs were positively validated by PCR assays, thereby confirming the reliability of the assembly.

**Conclusions:**

A first generation BAC-based physical map of half-smooth tongue sole was constructed with high reliability. The map will promote genetic improvement programs of this fish, especially integration of physical and genetic maps, fine-mappings of important gene and/or QTL, comparative and evolutionary genomics studies, as well as whole genome sequence assembly.

## Background

Half-smooth tongue sole (*Cynoglossus semilaevis* Günther) is a marine flatfish that belongs to the family Cynoglossidae in the order Pleuronectiformes, and is widely distributed in Chinese coastal water [1,2]. Because of its rarity and delicacy, half-smooth tongue sole has been exploited as a commercially important cultured marine fish, especially in the Shandong Peninsula [3]. Because female grows 2–3 times faster than male, the development of all-female stocks of this fish would be of significant benefit for aquaculture and this fish could be an ideal model for the study on sex-determination mechanisms in teleosts [4]. Genetic studies, especially on the sex-determining mechanism of this species, have been carried out in the last decade. The chromosomal sex-determining mechanism in half-smooth tongue sole was determined to be female heterogametic with the ZW chromosomes [5-7]. A large number of genetic markers [8-10], especially female-specific amplified fragment length polymorphism (AFLP) markers have been developed [4] and a large number of ESTs have been analyzed [11]. Recently significant progress on the development of gynogenetic stocks [6] and characterization of sex-related genes [12-14] has been made.

Half-smooth tongue sole has a relatively small genome of about 626.9 Mb as estimated by flow cytometry [15]. To study the genomics of half-smooth tongue sole, two bacterial artificial chromosome (BAC) libraries with an average insert size of 156 kb have been constructed previously [15]. Meanwhile, microsatellite-based genetic linkage maps with different densities have been constructed, and four quantitative trait loci (QTLs) related to growth rate, seven sex-related loci and five sex-related markers have been located on the relevant chromosomes [16,17]. These studies have laid the foundation for the future genetic improvement of half-smooth tongue sole. However, no genome-wide physical map has been constructed for half-smooth tongue sole to date, and there are still some difficulties in high-quality assembly of the next generation genome sequencing reads without assistance of a physical map, especially for the W chromosome of this fish due to abundance of repetitive sequences [18,19].

A physical map of a species is the starting point of the clone-by-clone genome sequencing approach [20] and always constructed as a series of linear orderings of clones in a genomic library using their overlapping. Map-based sequencing strategies are expensive and laborious and, as a result, they have partly been replaced by shotgun sequencing strategies [21]. However, abundance of repetitive sequences, large gene families and extensive segmental duplications always complicate the assembly of whole-genome shotgun reads obtained from next generation sequencing platforms, and only physical maps can deal with these problems [22,23]. Moreover, a genome-wide physical map is also one of the foundations for integration of physical, genetic and cytogenetic maps, and could be used to fine-map economically important genes and/or QTLs and evolutionary genomics studies [24-30]. The economically important genes and/or QTLs and genome sequence information of a species in agriculture are important foundations of marker-assisted selection breeding and whole genome selection breeding. Therefore, the construction of a physical map of the half-smooth tongue sole genome has become essential to complete the final whole-genome sequence assembly as well as to accelerate the progress in genetic improvement programs of this fish.

Several fingerprinting methods with BAC libraries had been developed, such as agarose gel electrophoresis, DNA sequencing electrophoresis and high information content fingerprinting (HICF) with SNaPshot labeling kit [31-33]. Each of them had been used to construct physical map for some species. For example, the physical map of human genome was constructed with the method of agarose gel electrophoresis [34], the physical map of soybean genome was constructed with the method of DNA sequencing electrophoresis [35], and the physical maps of wheat and *Brassica rapa* genomes were constructed using the method of HICF with SNaPshot labeling kit [29,36]. Now genome physical maps for a number of aquatic species have been also constructed with these different methods. For example, physical maps of threespine sticklebacks (*Gasterosteus aculeatus*) and Atlantic salmon (*Salmo salar*) were constructed with the method of agarose gel electrophoresis [37,38], physical maps of Nile tilapia (*Oreochromis niloticus*) and Zhikong Scallop (*Chlamys farreri*) were constructed with the method of DNA sequencing electrophoresis [39,40], while the method of HICF with SNaPshot labeling kit has been used to construct physical maps for channel catfish (*Ictalurus punctatus*), rainbow trout (*Oncorhynchus mykiss*), Asian seabass (*Lates calcarifer*) and common carp (*Cyprinus carpio*) [41-45].

Here we report a first generation BAC-based physical map of the half-smooth tongue sole genome constructed with the method of HICF and the FingerPrinted Contig (FPC) program v9.4 [46].

## Results and discussion

### BAC fingerprinting and data processing

**Figure 1:**
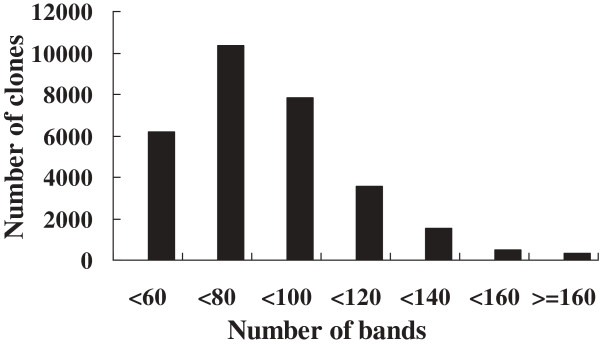
**The abundance distribution of restriction bands in BAC clones of half-smooth tongue sole (*Cynoglossus semilaevis*).** About 60% of the valid clones contained proper numbers of restriction bands ranging from 60 to 100 bands, and on average, one BAC clone contained 80.2 restriction bands.

The HICF method was chosen to develop a physical map of half-smooth tongue sole due to its well-established format with the commercially available SNaPshot kit (Life Technologies, Foster City, CA, USA) and its high throughput by using the ABI 3730xl sequencer (Life Technologies) [33,36,44,45,47]. A total of 33,575 clones (approximately 8.3-fold genome coverage) mainly from the *Hind*III BAC library were fingerprinted after digestion with a combination of five restriction enzymes. Of these fingerprints, 3,281 (9.8%) were removed by FPminer 2.1 (Bioinforsoft LLC, Beaverton, OR, USA) due to low quality. The remaining 30,294 valid clones (90.2%) represented approximately 7.5-fold coverage of the half-smooth tongue sole genome. The abundance distribution of the restriction bands in all these BAC fingerprints is presented in Figure 1. On average, each BAC clone contained 80.2 restriction bands, and about 60% of the valid clones contained proper numbers of restriction bands ranging from 60 to 100, and each band represented approximate 1.933 kb of BAC DNA fragment, as assessed from the average insert size (155 kb) of the *Hind*III BAC library [15].

### Determination of tolerance and cutoff

**Figure 2:**
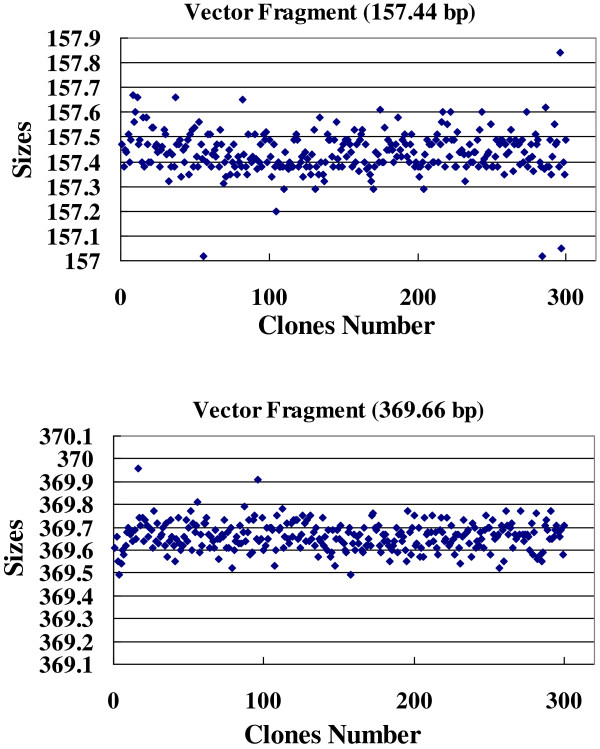
**The size distributions of two vector fragments in 300 randomly selected fingerprinting samples.** According to the 95% confidence intervals of the two vector fragments (157.4 bp and 369.6 bp), a tolerance of 4 was set for automatic contig assembly with program FPC, corresponding to 0.4 bp of primary fingerprint size.

Tolerance and cutoff are two important parameters used in the FPC program for contig assembly. Tolerance determines how closely two bands in different clones need to match to be considered as the same band. Its value could be set according to the observed size variations of particular bands in the project [42,44,48], and the size distributions of the two vector pECBAC1 fragments (157.4 bp and 369.6 bp) were analyzed (Figure 2). The standard deviations of them in 300 randomly selected clones were 0.085 bp and 0.062 bp, and the sizes of the 95% confidence intervals were 0.334 bp and 0.243 bp (Table 1). Because the FPC program does not allow the use of decimals and all fragment sizes were multiplied by 10, the tolerance value was set at 4, corresponding to 0.4 bp of primary fingerprint size. This value was first determined for SNaPshot-HICF by Luo *et al.*[33] and was used in construction of physical maps for several aquatic species [42,44,45].

**Table 1:** The standard deviations and 95% confidence intervals of two vector fragments in random 300 clones

**Vector fragments**	**Sample number**	**Standard deviation**	**95% confidence interval**	**Interval size**
157.44 bp	300	0.085 bp	157.271-157.604 bp	0.334 bp
369.66 bp	300	0.062 bp	369.540-369.782 bp	0.243 bp

Cutoff is a threshold of the probability that fingerprint bands of two clones match by coincidence. Lowering cutoff value could increase the stringency of contig assembly and decrease the probability of chimeric joining of duplicated or repetitive regions [42,44,45]. However, if cutoff value is set too low, some real BAC contigs will be split into small contigs or singletons. A series of preliminary tests were performed on the whole data with different cutoff values ranging from 1e-20 to 1e-75, and the observed changes in the numbers of questionable clones (Q clones), singletons, and contigs versus different cutoff values are presented in Additional file 1. Along with the decrease of cutoff value from 1e-20 to 1e-75, the number of singletons increased from 587 to 16259, the number of Q clones decreased from 12790 to 9. When cutoff value was 1e-60, the number of singletons was 8662 (28.6%) and the number of Q clones was 234 (1.1%). According to the method of *Brassica rapa* physical map assembly, the fraction of clones assembled (71.4%) was sufficient to give a robust basis for the further assemblies [36]. The very low fraction of Q clones shown that initial assembly with the cutoff value of 1e-60 was reliable. So a cutoff value of 1e-60 was reasonably stringent and chosen for the initial assembly.

### Contig assembly

The physical map contigs of half-smooth tongue sole were assembled using the FPC v9.4 program with a tolerance of 4 and an initial cutoff value of 1e-60 in three steps (Table 2). First, 4,200 contigs were constructed with 21,632 clones and 234 Q clones distributed in 145 Q contigs were produced in initial assembly. Then 39 Q contigs with more than 10% Q clones were broken up by “DQer” function. Finally, 2,715 contigs were end-merged by “End to End” function, and 8,077 singletons were added to the end of contigs by “keyset to FPC” function at nine successively higher cutoffs from 1e-60 to 1e-15. The average contig length was increased from 233 kb to 539 kb, and the length of the largest contig was increased from 958 kb to 3,481 kb, while the physical length of the total contigs was decreased from 980 Mb to 797 Mb, and the genome coverage was also decreased from 1.56 to 1.27. These changes suggested that the “End to End” and “keyset to FPC” functions of the FPC program obviously improved the quality of the contig assembly. The final physical map had 1,485 contigs assembled with a total of 29,709 BAC clones; 585 clones remained as singletons. A summary of the half-smooth tongue sole physical map data is presented in Table 3.

**Table 2:** The building process of half-smooth tongue sole physical map with 30,294 BAC clones

**Assembly Steps**	**Contigs**	**Singletons**	**Physical length(Mb)**	**Genome coverage**	**Q-contigs/Q-clones**	**Longest contig(kb)**	**Avr. contig length(kb)**	**NO. of contigs in different sizes**					
								**≥ 100**	**99-50**	**49-25**	**24-10**	**9-3**	**=2**
Initial 1e-60	4200	8662	980	1.56	145/234	958	233	0	1	28	493	2312	1366
DQer (1e-60 to 1e-87)	4260	8807	989	1.58	106/133	958	232	0	0	27	476	2365	1392
Merge 1e-55, 1	3792	7445	960	1.53	106/133	1030	253	0	3	40	604	2252	893
Merge 1e-45, 1	2845	5219	894	1.43	105/133	1465	314	1	7	140	742	1598	357
Merge 1e-35, 2	2592	3356	879	1.40	103/133	1465	339	1	11	198	779	1355	248
Merge 1e-25, 2	2022	1759	838	1.34	102/133	2221	414	4	46	267	735	844	126
Merge 1e-15, 2	1485	585	797	1.27	101/133	3481	539	9	83	311	598	427	57

Note: Contig assembly was performed with the tolerance of 4 and the initial cutoff value of 1e-60, followed by iteration of the end-merge, and singleton-merge routines by means of FPC v9.4. Additional end-merge and singleton-merge routines at 1e-40, 1e-30 and 1e-20 are not shown.

**Table 3:** Statistics of the first generation BAC-based physical map of half-smooth tongue sole genome

Number of BAC clones fingerprinted	33, 575	~8.3 × genome coverage
Valid fingerprints for FPC assembly	30, 294	~7.5 × genome coverage
Total number of contigs assembled	1,485	
Clones contained in the 1,485 contigs	29,709	~7.3 × genome coverage
Average number of clones per contig	20	
Average contig size in consensus bands (CB)	278	
Estimated average contig size (kb)	539	
Longest contig (ctg31; kb)	3,481	
Estimated N50 contig size (kb)	664	
Number of Q-contigs	101	6.8%
Number of Q-clones	133	0.45%
Number of singletons	585	1.93%
Average insert size of the BAC library (kb)	155	
Bands number of per BAC clone	80.2	
Average size each band represents (kb)	1.933	
Total number of bands included in the contigs	412,292	
Total physical length of assembled contigs (kb)	796,960	~1.27 × genome coverage

### Genome coverage

Based on the average insert size (155 kb) of the BAC library [15], the valid 30,294 clones summed up a total of 3,455.97 Mb, which represented 7.5-fold coverage of the haploid genome of half-smooth tongue sole. Though this coverage is smaller than the coverage reported for Atlantic salmon (11.5×) and rainbow trout (8.3×) [38,43], it is larger than the coverage obtained for Nile tilapia (5.6×), channel catfish (5.6×), Asian seabass (4.9×), common carp (5.9×) and Zhikong scallop (5.8×) [39,40,42,44,45]. Therefore, the 30,294 valid clones obtained here should be sufficient to construct a practicable and reliable BAC-based physical map of half-smooth tongue sole.

There were a total of 412,292 consensus bands in the final version of contig assembly, representing approximate 797 Mb of genome physical length (412,292 × 1.933 kb per consensus band). On average, each BAC clone contributed 13.9 distinct bands or 26.8 kb linear length to the assembly. The estimated physical length was slightly longer than the size of half-smooth tongue sole genome estimated by the flow cytometry method (626.9 Mb) [15], and was about 1.27× genome coverage. The longer genome coverage might be due to the overestimation of the average insert size of the BAC library, or the heterogeneity of BAC DNA from three female half-smooth tongue sole fishes. Similar results were reported for the physical maps of other species such as Nile tilapia (1.65×), Zhikong scallop (1.5×) and turnip (1.3×) [36,39,40]. This result also implied that the resultant contigs did not sufficiently overlap with each other and the gaps between the contigs might be closed by additional BAC fingerprints or additional rounds of end-merging at lower stringency [49]. Lower stringency, however, would likely decrease the reliability of the resultant physical contigs and should be performed by manual editing with the assistance of markers.

### Q clones and Q contigs

If a clone contains more than 50% extra bands, which do not actually align to the map, it would be labeled as a Q clone even when it is correctly located on the consensus map [46]. Q clones are generated by inconsistency in enzyme digestion, cross-contamination, abundance of repetitive sequences, and/or extensive segmental duplication, even genome duplication, and its existence could result in a false positive overlap with another clone and even with another contig. Q contigs, especially those with more than 10% Q clones, are usually broken into two or more contigs to prevent or decrease the chance of chimeric joining. In this study, the initial contig assembly with a cutoff value of 1e-60 produced only 41 Q contigs having more than 10% Q clones. The “DQer” function was performed by decreasing the cutoff value as low as 1e-87 where necessary and broke up 37 Q contigs with more than 10% Q clones. Although the numbers of contigs and singletons increased, the reliability of the resultant contigs should be greatly improved.

In the final version of assembly, there remained a total of 133 Q clones distributing in 101 Q contigs, corresponding to 0.45% of the clones assembled in the physical map. This fraction is much less than the fractions of Q clones reported in the physical maps of other species such as channel catfish (4.3% and 7.3%), rainbow trout (1.4%), Asian sea bass (4.6%), common carp (2.1%), maize (11%) and turnip (15%) [36,41-45,49]. The fraction of Q contigs (6.8%) was also lower than the fraction reported in the maps of other species such as Nile tilapia (24.3%), channel catfish (15.6%), rainbow trout (19.4%) and common carp (23.9%) [39,42,43,45]. These fractions implied that the chance of false positive overlap in our assembly was substantially lower than these species.

Nelson *et al.*[49] demonstrated that most increase of the number of Q clones in a map mainly came from the adding of singletons into the ends of contigs. However, in our study, the number of Q clones did not increase along with the integration of the 8,077 singletons into the ends of contigs and remained invariable at the very low level of 133. This finding also suggested that the most of singletons merged in the end of the contigs were from the regions of low coverage.

### Size distribution of contigs

**Figure 3:**
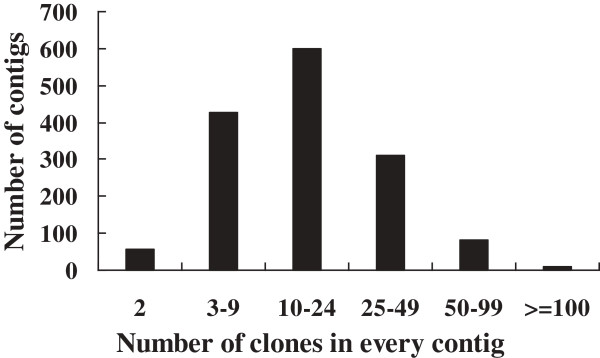
**The size distribution of contigs in the half-smooth tongue sole genome physical map.** 96.2% of the contigs contained more than 2 BAC clones, 67.4% contained more than 9 clones and 27.1% contained more than 24 clones. On average, each contig contained 20 BAC clones.

With the increase of cutoff value from 1e-60 to 1e-15, the average size of the contigs was also increased (Table 2). The final size distribution of contigs in the physical map of the half-smooth tongue sole genome is shown in Figure 3. Overall, most of the contigs (96.2%) contained more than two BAC clones, 67.4% contained more than nine clones, and 27.1% contained more than 24 clones. On average, one contig in the final assembly contained 20 BAC clones. The largest contig (ctg31) contained 410 BAC clones and had a physical length of 3.48 Mb. The N50 length of the final assembly was 664 kb and there were 394 contigs longer than it. These contigs would be a useful resource for the future integration with linkage map and whole genome sequence assembly.

### Assessment of the physical map

The half-smooth tongue sole physical map assembly was judged to be reliable preliminarily based on enough genome coverage of valid clones, the very low cutoff value, and the very low fractions of Q clones and Q contigs. PCR assays of randomly selected contigs were used to further assess the reliability of the physical map assembly. If all clones of a contig truly overlap and belong to the contig, they should be identified by PCR amplification with proper primer pairs, thereby validating the contig [45]. Twenty-one contigs of various lengths (307–1276 kb) were randomly selected with consideration of the distribution of the clones end-sequenced. The average length and clone numbers of these contigs were 530 kb and 32, respectively. For short contigs, PCR reactions were performed on all clones of each contig, while for long contigs, reactions were conducted on some near the clones developing primers.

**Figure 4:**
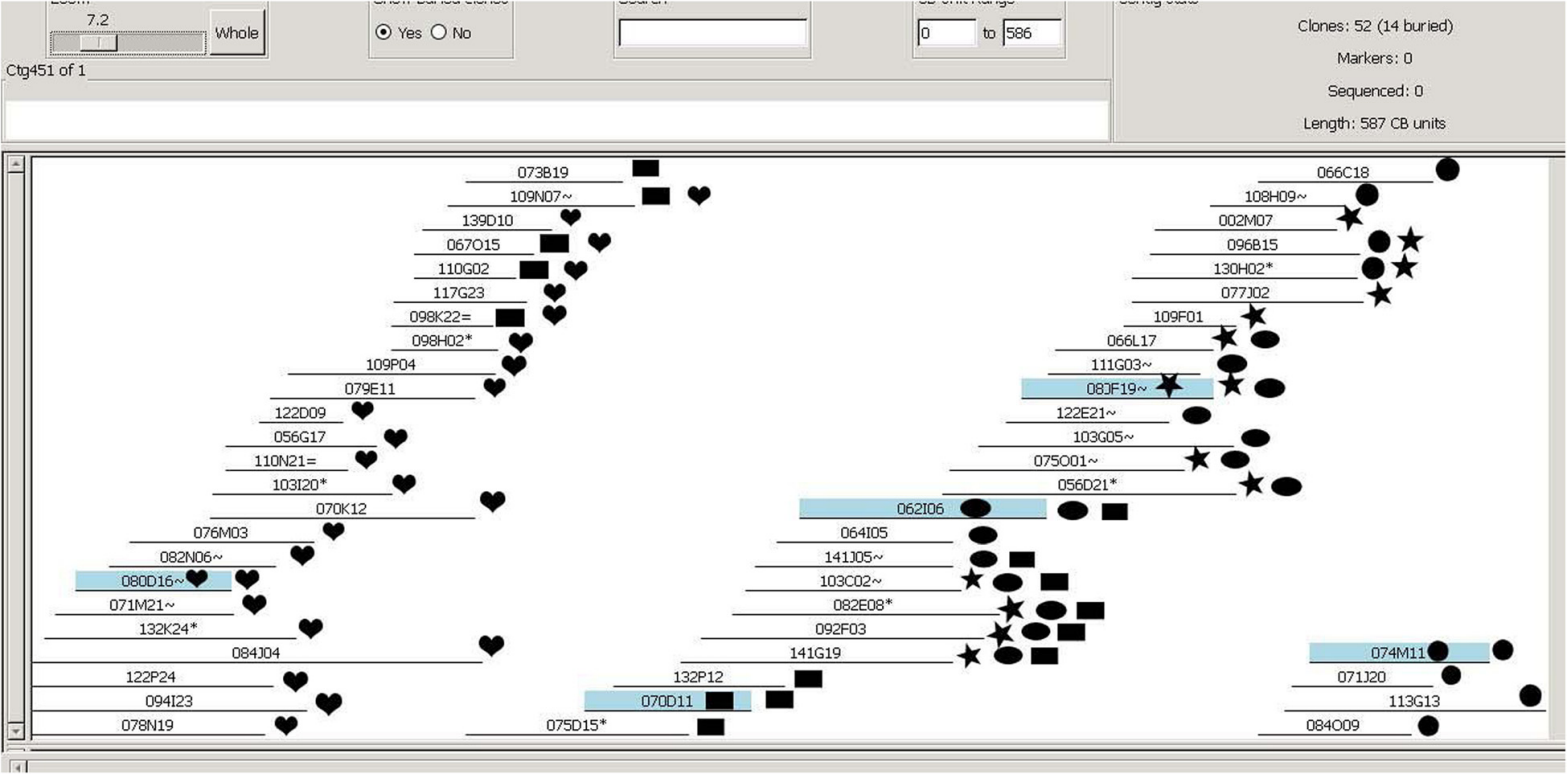
**A contig positively identified using PCR assays with five pairs of BES-based primers.** BAC clones positively amplified are indicated on the right with shaped symbols same to the symbols marked on the clones developing primers. The five pairs of primers collectively identified all clones positively, allowing confirmation of the contig.

The results of the PCR assays of 21 contigs are shown in Table 4. All clones of 20 contigs were positively identified by the PCR assays with one or multiple pairs of primers, respectively, and an example (ctg 451) is shown in Figure 4. Fifty-two clones were included in the contig, and five pairs of PCR primers were developed from the end sequences of five clones (080D16, 070D11, 062I06, 080 F19, 074 M11). All clones near each of these five clones were positively amplified, respectively, and finally all of the 52 clones were positively identified. But in ctg143, one clone (141O09) could not be positively identified by this way. This negative result might arise from either the lack of proper primers or possible chimeric overlapping during the assembly process. Overall, 675 of the 676 BAC clones (99.9%) in the 21 contigs were positively validated, confirming the high accuracy and reliability of the assembly.

**Table 4:** Assessment of the reliability of randomly selected 21 contigs with PCR assays

**Contig ID**	**Physical lengths (kb)**	**Number of clones**	**Number of positive clones**	**Proportion of positive clones**	**Number of primer pairs**
724	360	18	18	100%	3
2,113	383	17	17	100%	3
27	309	16	16	100%	2
2,259	307	13	13	100%	2
9	499	28	28	100%	2
17	410	19	19	100%	2
85	479	20	20	100%	2
122	352	21	21	100%	2
195	501	42	42	100%	3
148	431	16	16	100%	2
172	397	34	34	100%	2
14	396	43	43	100%	4
1,458	462	27	27	100%	3
52	617	34	34	100%	3
175	704	51	51	100%	2
996	329	43	43	100%	1
252	503	26	26	100%	4
143	941	63	62	98.4%	7
113	1,276	76	76	100%	9
451	1,135	52	52	100%	5
26	360	17	17	100%	2
Total	11,128	676	675	99.9%	65
Average	~530	~32	~32		~3

## Conclusion

A first generation BAC-based physical map of the half-smooth tongue sole genome was constructed with 30, 294 valid fingerprints (7.5 × genome coverage) using the method of HICF with SNaPshot kit and the FPC program v9.4. A total of 29,709 BAC clones were assembled into 1,485 contigs with an average length of 537 kb and a N50 length of 664 kb. The physical length of the assembled map was 797 Mb. The reliability of the map assembly was validated by PCR assays on randomly selected 21 contigs. This physical map will promote the assembly of W chromosome and genetic improvement of half-smooth tongue sole.

## Methods

### Ethics Statement

All the experimental procedures involved in this study were approved by the Yellow Sea Fisheries Research Institute's animal care and use committee, and followed the experimental basic principles.

### Source of the BAC library

Two BAC libraries, in the *BamH*I and *Hind*III sites of the vector pECBAC1, were developed previously from three female half-smooth tongue sole fishes in our laboratory. The two libraries were arrayed in 144 384-well microtiter plates and consisted of a total of 55,296 BAC clones with an average insert size of 156 kb, representing 13.4-fold coverage of the haploid genome [15]. Nearly all the BAC clones used for fingerprinting in this study were from the *Hind*III library with an average insert size of 155 kb. A few of the BAC clones were from the *BamH*I library.

### BAC DNA isolation and fingerprinting

To decrease the differences in BAC DNA yields, the BAC clones were inoculated and precultivated in new 384-well plates containing 60 μl of 2 × YT medium plus 12.5 μg/ml chloramphenicol using a 384-pin replicator (BOEKEL, Feasterville, PA, USA). Plates were covered with adhesive air permeable seals (Excel Scientific, Victorville, CA, USA) and incubated at 37°C for 21–22 h with shaking at 300 rpm. Then the precultivated BAC clones from each 384-well plate were inoculated into four 96 deep-well plates using a 96-pin replicator (BOEKEL, Feasterville, PA, USA). Each well contained 1.4 ml of 2 × YT medium plus 12.5 mg/ml chloramphenicol. The 96-well plates were covered and incubated at 37°C for 24–26 h with shaking at 300 rpm. BAC DNA was isolated using a modified alkaline lysis method followed by purification with 70% ethanol [50]. Dried BAC DNA was resuspended in 35 μl ddH_2_O and stored at −20°C before use.

BAC DNA fingerprinting was performed according the method of Luo *et al.*[33]. The DNA of each clone was digested with a mixture of five restriction endonucleases, *BamH*I, *EcoR*I, *Xba*I, *Xho*I, and *Hae*III (New England Biolabs, Ipswich, MA, USA) at 37°C for four hours. Fragments were end-labeled with the SNaPshot kit at 65°C for 60 minutes. The labeled BAC fragments were precipitated with sodium acetate and pre-chilled 100% ethanol following by washing with 70% ethanol. Dried DNA fragments were resuspended in 10 μl of Hi-Di formamide plus 0.05 μl GeneScan-500 LIZ as an internal size standard, denatured for 5 min at 95°C, and analyzed on a 3730xl DNA Analyzer (Life Technologies).

### Fingerprint collection and processing

The fragment sizes of all BAC clones were collected by the Data Collection program on the ABI 3730XL Genetic Analyzer, and then processed with FPminer 2.1 software. Briefly, the threshold in peak finding was set as 35 relative fluorescent units (RFU), and the size range was set as 50–500 bp. Fragments with a peak height greater than 6,000 RFU or with width greater than 15 were removed. Automatic fingerprinting editing was used to remove the potential background, and 70% was set as the cutoff percentage for the blue channel, while 75% was set for the other three color channels. The average highest peak in every color channel was counted from the top 3rd to the 7th peaks. After a cross-contamination check, potential contaminated clones with similarity coefficients greater than 0.25 were removed. All samples with a Size Standard Matching Quality Score below 0.9 or with a Fingerprint Editing Quality Score below 10 were also removed. In addition, vector and potential repetitive DNA fragments with frequencies greater than 20% were identified and removed by fragment frequency analysis. Lastly, only the size files with 30–200 fragments were exported for contig assembly with the FPC program.

### BAC contig assembly

Physical map contig assembly was performed with the FPC program v9.4 (http://www.agcol.arizona.edu/software/fpc) [46]. The FPC parameters were adjusted for the method of HICF as described by Nelson *et al.* and Xu *et al.*[42,48]. The standard deviations of the size distributions of two vector fragments (157.4 bp and 369.6 bp) in 300 randomly chosen BAC clones were calculated to obtain the 95% confidence intervals. The tolerance value was set to 4 according to the result of these calculations, and the gel length was set at 18,000 bp in consideration of the size range (from 50 bp to 500 bp). Because the average insert size was 155 kb and the average valid bands were 80.2 per clone, the average size per band was estimated to be 1,933 bp. The “Best of” function was set to 100 builds. Then a series of preliminary contig assemblies to determine the optimal cutoff value, which would limit the number of Q clones and avoid a great decrease of genome coverage, was performed on the whole data with different cutoff values ranging from 1e-20 to 1e-75. Based on the results of these tests, a very low initial cutoff value of 1e-60 was chosen to carry out the initial contig assembly. Contigs with more than 10% Q clones in initial assembly were broken up by the“DQer” function with a step size of 9. Then, the stringency was decreased at nine successively lower cutoff from 1e-60 to 1e-15. At each step, the “Ends to Ends” auto merge function was used to merge the resulting contigs with a minimum of one (from 1e-60 to 1e-45) or two (from 1e-40 to 1e-15) matching ends and the “keyset to FPC” function was used to merge the singletons to the end of the contigs, respectively.

### Physical map quality assessment

Physical map quality assessment was performed using PCR assays as described by Xu *et al.*[45]. The contigs to be assessed were selected randomly, but the even distribution of the clones end-sequenced in contig was also taken into consideration so as to develop enough and appropriate PCR primers. All clones in the selected contigs were inoculated from the stocking 384-well plates. BAC DNA was extracted using the alkaline lysis method as described above. All of the primers used in the assays are listed in the table of Additional file 2. Twenty-five μl of PCR solution contained 1 × PCR buffer, 160 μmol/L of each dNTPs, 0.12 μmol/L forward primer, 0.12 μmol/L reverse primer, 1 mmol/L MgCl_2_, 1 U of Taq DNA polymerase (Fermentas, Glen Burnie, Maryland, USA) and about 10 ng BAC DNA. Reactions were conducted on all or some of the BAC clones in a specific contig under the following conditions: initial denaturation at 95°C for 5 min; then 35 cycles of 95°C for 30 s, 55°C for 30 s and 72°C for 30 s; and final extension at 72°C for 5 min. The PCR products were then subjected to electrophoresis on 1.2% agarose gel. The BAC clones that produced specific bands with proper sizes were considered to have true overlap with the clones used to develop primers.

## Competing interests

The authors declare that they have no competing interests.

## Authors’ contributions

JZ worked on clone culture, data process, map assembly and drafted the manuscript. CS participated in the study design, library manipulation and provided end sequences. LZ worked on DNA extraction and purification. KL participated in enzyme digestion and fluorescent labeling. FG participated in data collection. ZD participated in contig validation. PX guided the experiment and provided assistance for data analysis and manuscript preparation. SC conceived, designed and supervised the entire study. All authors read and approved the final manuscript.

## Supplementary Material

Additional file 1 **The observed changes in the numbers of Q clones, singletons, and contigs versus cutoffs.** A series of preliminary assemblies of half-smooth tongue sole physical map were performed on the whole data with different cutoff values ranging from 1e-20 to 1e-75. A cutoff value of 1e-60 was chosen for the initial automatic assembly. Click here for file

Additional file 2 List of primers used for assessing the half-smooth tongue sole physical map. Click here for file
