# Supplementary material for: A first generation BAC-based physical map of the half-smooth tongue sole (Cynoglossus semilaevis) genome
Source: BMC Genomics. 2014 Mar 20;15:215. doi: 10.1186/1471-2164-15-215 (PMC3998196; doi:10.1186/1471-2164-15-215)
Supplement: Additional file 1 — The observed changes in the numbers of Q clones, singletons, and contigs versus cutoffs. A series of preliminary assemblies of half-smooth tongue sole physical map were performed on the whole data with different cutoff values ranging from 1e-20 to 1e-75. A cutoff value of 1e-60 was chosen for the initial automatic assembly. [file 1471-2164-15-215-S1.doc]

**Additional file 1**

**The observed changes in the numbers of Q clones, singletons, and contigs versus cutoffs.** A series of preliminary assemblies of half-smooth tongue sole physical map were performed on the whole data with different cutoff values ranging from 1e-20 to 1e-75. A cutoff value of 1e-60 was chosen for the initial automatic assembly.
