# Supplementary material for: A first generation BAC-based physical map of the half-smooth tongue sole (Cynoglossus semilaevis) genome
Source: BMC Genomics. 2014 Mar 20;15:215. doi: 10.1186/1471-2164-15-215 (PMC3998196; doi:10.1186/1471-2164-15-215)
Supplement: Additional file 2 — List of primers used for assessing the half-smooth tongue sole physical map. [file 1471-2164-15-215-S2.rtf]

Additional file 2
List of primers used for the assessment of Half-smooth tongue sole physical map.
Contigs	Primers	Forward primers	Reverse primers	
Contig724	065E22F	CGCCAGGGCTTTTAAATACTCT	AACCGAGGACCAGAAACAAGTA	
Contig724	066K02F	GAAATGATTCCAATGCCCTGAG	GCTCCAGGCATTTAATCAGG	
Contig724	066K02R	TGGTGGCTGATTTATTTCCCG	GGTTTGAACTGATTGCTGTGGT	
Contig2113	082O20F	TGAAGCCCAGGAGCATTCAC	GCAACCAGGTCATTACATCAGC	
Contig2113	067G07R	TTAATCTGACAGGCATCGCACT	GCTTCTTCTCTCCGTCTCTATCT	
Contig2113	079O04F	TCCTAAAGTCGTCCACACCC	AAAGCCTTCCACCGTGACA	
Contig27	066D01F	GACGACAGACAGAAGAATGAGC	ACCAGAAACGCCGCAACAT	
Contig27	066D01R	TAAAGCACAACAGGCACCCA	GTACTGCACCGAGGCTGGT	
Contig2259	084A21R	AGGAGTTGGTGGTGAGTGGT	GGAGTCTCTGTTTATTCGCCTC	
Contig2259	081M01R	TCCTGAAGCAGCCGAGAAAT	TCAACCAAGACAGACCCACA	
Contig9	066N24F	AAGTCACATCCACTGAGCCG	AGCCATCCGCTACATAAGCC	
Contig9	066N24R	ACTTCCTGCCTCACCTAAGC	ACCAAACAGACCTCACAAAGG	
Contig17	065D21R	GTGACTCAACAACACCCTCCC	ACAGATGATTCAGGGTCAGGTC	
Contig17	083K07R	GATGAATCGGAGCGACACG	ACCCGTTGATAAAGTTTGAAGACAC	
Contig85	075B22F	TTTGCTCCTGCTGACATCCT	AGCCACCGAGTAATGAACACT	
Contig85	075B22R	CTAAAACAGCCAGTAGTATGCCAC	TGTAGAGACTGCACGCATAGATC	
Contig122	074O09F	AACCGCACGACAACTTTGG	ACCCTACACAGAGAGCCAGA	
Contig122	074O09R	GCACCAGTAGATTTAGGAGACCCAG	ATTGGTGACCCGTCCAGAACAGA	
Contig195	069I18F	GCAAGTGACATAGACGCAGC	AAGGAAAGTGGGACAGGGAG	
Contig195	069D12F	CTATCATCTCACTCCTCCTCCTT	CAGTTTGGTTTCTCTCCTCCCT	
Contig195	069D12R	ATCATGATGAATTCTGTGTTGGG	GAGGATGTAATGGTTGTGTAGGC	
Contig148	075F01 F	AGTGTTGGCTCTTGTGACCA	GACAGGTAGGCTGGGAGAAAG	
Contig148	080O08R	GCAGCAGCGACTTACTCTG	CACTCATCACTTGGCACTTTGT	
Contig172	066F19F	CCTGCCCTGGTTGTTGTCA	CACAGTGACGATGTGGAGACAG	
Contig172	066F19R	TAAACAGGTTGAATCGCAGACA	GAAGAAATTACCGAGTGGAACAAC	
Contig14	069N13R	ACAGCAGTGAGAACAAGAATCC	GCCAGCGAACAAGAAGAGTAG	
Contig14	085L13F	CCAAACCCAAACAGAGACAGG	ACCAGTCACCAAGGACCCTA	
Contig14	085L13R	CAACCGTGGAGAAATAACCTTCA	AGGGAGCAACAAGCAAGTTTAC	
Contig14	075M24F	AACAGAAAGACAAACACGAGCAAG	GTTAAAGATGCACAGTGATGAC	
Contig1458	066N23F	TTGCCCTTCTTTAGACACTCTC	TGCATGTCAGTGTGGGTGG	
Contig1458	066N23R	CTGCCCAAACAAGTCCTGC	GCTGCTTTCTGCCTCCTTAG	
Contig1458	069D06F	GATTGGTCACCTGCTACTCCC	AGAAGTGATGCTTGCAGTGGT	
Contig52	064D16R	CAACCATACTTTCCCAGAAGGT	GTTCACTTTCCACAGCAGGAG	
Contig52	085G16F	TGGTCGTTTCGGACTCTTGG	GAGACAGATGACAAAGCACCTC	
Contig52	085G16R	GACCTGATAGACCCTCGCACT	AGAGCCGTTCCTGTGAGCA	
Contig175	070B21R	ATGTAGTTGTTGGAAACCAGGG	AAGCCACGGAACCAGAGATG	
Contig175	075B10F	AAACCAACTGTGGAGGAGGC	GGACAGCAGTATAAAACAAAGCAG	
Contig996	069P13F	TCCAGAGGCAGAGTGGGTT	GTGGGTTGTTCTTTCAGTTACCT	
Contig252	066I01R	GTCCGTCTCGTGTGGACCT	GCTGCGTTTGTGGTAAATTGC	
Contig252	079N18F	CATGCCAAGAACATCATTCACC	CCTCCTTCGATTATAGTGCTGTG	
Contig252	079N18R	CGCAGAACCAGGATTAAAGTCT	TCCCTGCTTGTACCTCATTAGTTA	
Contig252	084G22F	CGGACAGCCGCATCAACAA	CGGGAGAGCTTTGAACAGGT	
Contig143	074G21F	GAGTTTGTATTCCACTGAGCGG	TCCTCCACCATCTTCGTATGC	
Contig143	062J23F	ATTTGCCACTAAGCACGCC	TCACACACATACAGCAGATGG	
Contig143	062J23R	CTGACTTTGCTGGTGTGACG	TGTAGAGTTTGGGACTGGGC	
Contig143	084M14F	TCCGTGCCACTCTTCTTACAG	ACACTGGAACCACACAAATGC	
Contig143	084M14R	GTGAAATGAGAGGAAGGGAAAGC	TCAGGAAATAGAGCAGAGCAGG	
Contig143	080H08F	TGAATGCAGAAAGTGGCAGG	GTGCCGTGAGAGATCATTAGGT	
Contig143	080H08R	GTGAGAATGGAGGTCTTGCGT	AGAACTGAGAACAGAGGCTAATCT	
Contig113	085K12R	GTGCCTTTCACACTATGGCTT	CTGGTCACAAACACTCCTTGG	
Contig113	085L08F	AAGAGGAAGTGAGCCAGTGATAA	AGTTGAAAGAAGAAGGTGCCAC	
Contig113	074E15R	CTGTCACCTCATTGGTTTGTGG	AGAGCCTTCACCTATTTCCAGT	
Contig113	068D17R	GACCAAAGGGAGAAATGAGTCC	CTGGACCCAAACAAAGATCCTT	
Contig113	067L08F	GACCAGACAGAGCAAAGCAA	AGCAGCCACAGGAACCAGT	
Contig113	067L08R	ATCTGTTGTACTTGTTGCCCG	TCCTCCTGTCAATCAACCTGG	
Contig113	070F24F	CTCCTCCATATCTTTCAACCATCA	CAACTTGGTGTATCTGACCAGC	
Contig113	085E13R	CTCACTAACTGTCCAATAACACGG	TGTAAACACAAACCACCCGC	
Contig113	069J19R	GGTAGAAACAAACATTACAGGTGG	TGGAGATGGATGGGAGAGGAT	
Contig451	074M11F	GGCAATAAAGAGCAGAGTAGACC	AACACAACTAAAGCCCACCTG	
Contig451	084O09F	AGCAGTCCACAAAGACGAGT	TGCGGAGACCATCAAGAGAAT	
Contig451	062I06F	TGGCAGTCGGTATCAGGAGT	GCAAGTAGGTCAGCAGAAAGAG	
Contig451	070D11F	ACAGTGGAAGGAGGCGAAG	CAGAGACACGGCAGGAAGT	
Contig451	080D16R	GCAGGTTTCTGTTCTCTCTGAG	CCAGTGCATCTCCATGTCATT	
Contig26	067M19R	AAAGCACATACCTGATTTAGCC	GAAACAAGACTTGAAACAAACGAC	
Contig26	062G01F	CTATGTGGAGGCAGTAAATGTCA	GTGTAAGCCAGTTAGGGCAC	
